# Electrophysiological Consequences of Cardiac Fibrosis

**DOI:** 10.3390/cells10113220

**Published:** 2021-11-18

**Authors:** Sander Verheule, Ulrich Schotten

**Affiliations:** Department of Physiology, Cardiovascular Research Institute Maastricht, Maastricht University Medical Center, 6200 MD Maastricht, The Netherlands; schotten@maastrichtuniversity.nl

**Keywords:** fibrosis, conduction, arrhythmias, tissue structure, heart

## Abstract

For both the atria and ventricles, fibrosis is generally recognized as one of the key determinants of conduction disturbances. By definition, fibrosis refers to an increased amount of fibrous tissue. However, fibrosis is not a singular entity. Various forms can be distinguished, that differ in distribution: replacement fibrosis, endomysial and perimysial fibrosis, and perivascular, endocardial, and epicardial fibrosis. These different forms typically result from diverging pathophysiological mechanisms and can have different consequences for conduction. The impact of fibrosis on propagation depends on exactly how the patterns of electrical connections between myocytes are altered. We will therefore first consider the normal patterns of electrical connections and their regional diversity as determinants of propagation. Subsequently, we will summarize current knowledge on how different forms of fibrosis lead to a loss of electrical connectivity in order to explain their effects on propagation and mechanisms of arrhythmogenesis, including ectopy, reentry, and alternans. Finally, we will discuss a histological quantification of fibrosis. Because of the different forms of fibrosis and their diverging effects on electrical propagation, the total amount of fibrosis is a poor indicator for the effect on conduction. Ideally, an assessment of cardiac fibrosis should exclude fibrous tissue that does not affect conduction and differentiate between the various types that do; in this article, we highlight practical solutions for histological analysis that meet these requirements.

## 1. Types of Fibrous Tissue and Fibrosis

**Figure 1:**
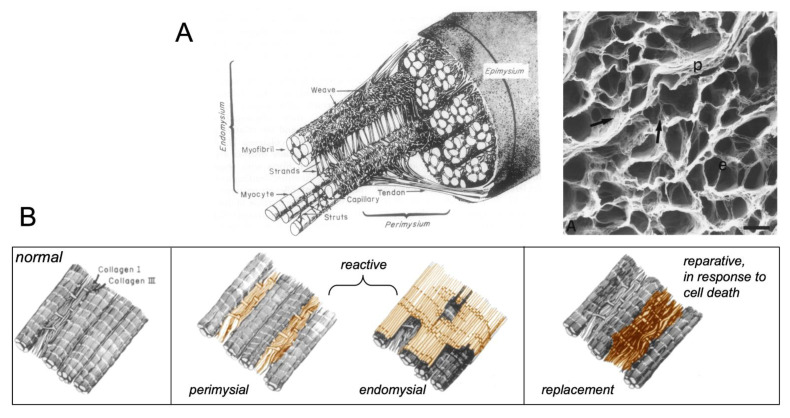
Fibrous skeleton of the heart and patterns of fibrosis. (**A**) Schematic illustration of the normal collagen matrix, illustrating perimysial fibrous tissue enveloping myocyte bundles and endomysial fibrous tissue in between strands of myocytes within bundles (left panel). Acellular preparation after digestion of cells showing perimysial sheets (p) and endomysial septae (e). (**B**) Schematic representation of the normal ECM (left panel), endomysial and perimysial fibrosis (middle panel), also called reactive fibrosis, and replacement/ reparative fibrosis secondary to myocyte death (right panel), adapted from [1].

Fibrous tissue forms the skeleton of the heart, providing the myocardial stiffness and mechanical stability required for normal cardiac function (Figure 1). In the healthy heart, different types of fibrous tissue can be distinguished. On the epi- and endocardial side, the myocardium is shielded by contiguous layers of epicardial and endocardial fibrous tissue. Larger coronary blood vessels are surrounded by perivascular fibrous tissue. Bundles of myocytes are ensconced by perimysial fibrous tissue. Within these bundles, endomysial fibrous tissue forms thin collagenous septa between (strands of) myocytes [1,2]. Fibrosis is a pathological increase in the amount of fibrous tissue. In the heart, both normal and excess fibrous tissue consist primarily of the fibrillar collagen types I and III [3]. All the types of normal fibrous tissue mentioned above can increase in volume in response to pathological conditions. For example, perivascular fibrosis occurs in conditions of pressure overload, hypertension, and heart failure [4,5]. The thickness of the epicardial fibrous layer (mainly by fibrin deposition) can rapidly increase as a result of tissue manipulation, for example, during epicardial mapping procedures. Ageing, increased stretch, and RAAS activity lead to endomysial and perimysial fibrosis [2]. Because of this, perimysial and endomysial fibrosis have also been called ‘reactive fibrosis’. This is distinctly different from reparative or replacement fibrosis, which is the final stage of the wound healing process after myocyte apoptosis or necrosis, most notably after myocardial infarction (Figure 1) [2].

**Figure 2:**
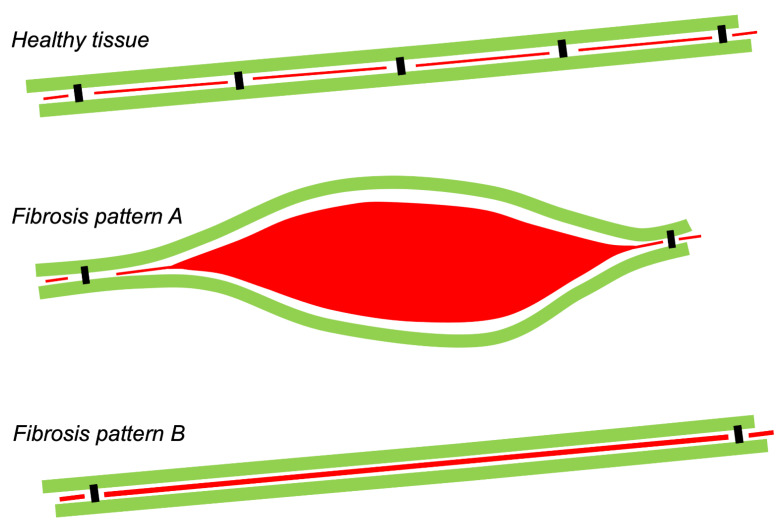
Relation between amount of fibrous tissue and electrical consequences. Conceptual diagram of two strands of longitudinally coupled myocytes (green) connected by sparse, discrete transverse connections (black). In between transverse connections, myocytes are separated by (endomysial) fibrous tissue (red). In fibrosis pattern A, a large area of fibrous tissue separates the strands, and in pattern B, the thickened endomysial septum. Both patterns have reduced transverse connectivity by the same degree, and will therefore have similar consequences for propagation, although pattern B is detected more readily in a histological analysis.

Current knowledge on the risk factors and signaling pathways underlying the development of fibrosis have been expertly reviewed elsewhere (e.g., in reference [3].) This review deals with the relation of fibrosis to electrical propagation. In general, the impact of fibrosis in arrhythmogenesis is governed by the extent to which electrical connections between myocytes are disrupted, and the distribution pattern of these lost connections. As depicted in the schematic form in Figure 2, the degree of lost connectivity may not agree with the amount of fibrous tissue, as observed histologically. Therefore, to understand the effect of fibrosis on propagation, it is important to first consider normal connections between myocytes.

## 2. Electrical Connections and Propagation

**Figure 3:**
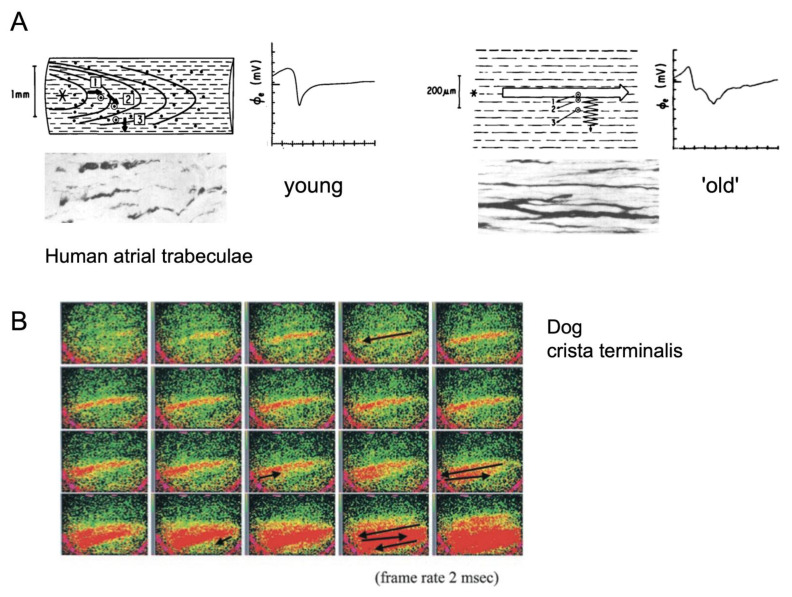
Non-uniform anisotropy of conduction. (**A**) Comparison of the conduction patterns and electrograms during pacing in human atrial trabeculae from a young and older individual (top panels). Electrograms recorded at locations transverse to the main wavefront. The corresponding histological illustrations show thickened collagenous septae in the older trabecula, adapted from [12]. (**B**) Illustration of conduction in the terminal crest of an older dog, showing zig-zag propagation of a narrow activation wave (red), conducting discontinuously in the transverse direction, adapted from [7].

Propagation of the cardiac action potential is mediated by gap junction channels [6]. In neonatal working myocardium, gap junctions are localized around the entire periphery of the myocytes. With further postnatal maturation of the heart, myocytes become increasingly polarized, with numerous large gap junctions at the intercalated discs at end-to-end connections, and smaller gap junctions at side-to-side connections [7]. The ability to form transverse connections also depends on the lateral separation of myocytes, which is determined by collagenous septa (endomysial and perimysial fibrous tissue). The thickness and length of these septa show regional differences in the healthy heart. In regions with a strong preferential fiber orientation, these septa tend to be longer. For example, in Bachmann’s bundle, the main conducting pathway between the right and left atrium, Spach et al. have shown, based on a careful reconstruction of tissue architecture, that perimysial septa can separate strands of myocytes over a length of 1–2 mm [8]. Macroscopically, the anisotropy ratio in conduction velocity is 3–6 in regions with a strong preferential fiber organization, such as the right ventricular trabeculae [9] and Bachmann’s bundle [8], and close to 1 in regions with a lower degree of organization, such as the atrial free walls [10]. At a more microscopic level, Spach et al. have shown that in small atrial bundles, transverse propagation has a discontinuous character, with delays between the action potential upstrokes in adjoining myocyte bundles [11,12,13]. With increasing age, transverse separation by collagenous septa becomes more pronounced, with a concomitant increase in discontinuous transverse conduction (Figure 3) [7,12].

**Figure 4:**
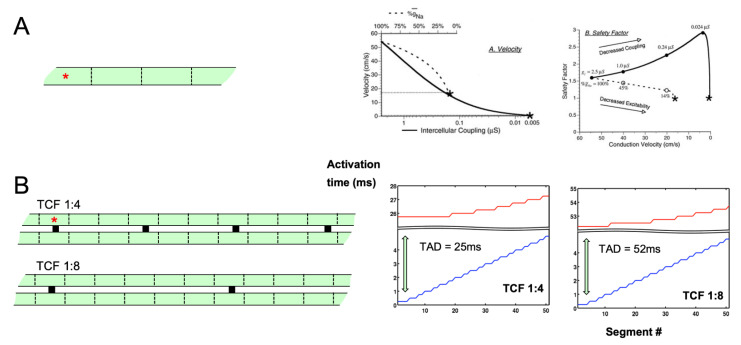
Characteristics of longitudinal and transverse propagation. (**A**) Linear strand of longitudinally coupled myocytes (left panel). When the sodium current was reduced, propagation velocity decreased by about 60% when conduction block appeared (middle panel, dashed line). Until that point, the safety factor for conduction decreased monotonically. When gap junctional coupling was reduced, the conduction velocity could be reduced to much lower values before the block occurred (middle panel, solid line). Under these conditions, the safety factor initially increased, and the action potential upstroke became increasingly dependent on the calcium current, adapted from [14]. (**B**) Two strands of longitudinally coupled myocytes connected by discrete transverse connections every fourth (1:4) of eight (1:8) elements. The paced upper strand activated rapidly (right panels, blue lines), while the non-paced lower strand activated within a very short period (right panels, red lines), but after a long transverse activation delay (TAD), which was highly sensitive to the number of transverse connections. Under these conditions, the action potential upstroke in the non-paced strand was still sodium-dependent, but the calcium current in the paced strand was required to maintain the plateau in the paced strand over the long duration of the TAD, adapted from [17]. Red asterisks indicate pacing sites.

The differences in nature between longitudinal and transverse propagation have also been studied in simplified mathematical models. In a linear strand of myocytes with longitudinal connections, Shaw and Rudy studied the effect of sodium current availability and gap junctional coupling. In a linear strand, a reduction in sodium current causes a moderate reduction in conduction velocity before conduction block occurs [14]. However, a reduction in gap junctional coupling led to very slow conduction with a high safety factor (i.e., conduction block is unlikely to occur). Under these conditions, the slow upstroke of the action potential is carried primarily by the calcium current, rather than the sodium current. This paralleled earlier findings in myocyte pairs by Joyner et al. [15], and in cultures of neonatal myocytes by Kleber et al., where gap junctional uncoupling led to a paradoxical decrease in the occurrence of conduction block [16]. We have reported a different type of behavior for transverse propagation in a mathematical model of two parallel strands of myocytes (Figure 4) [17]. When one strand was stimulated, the other strand could be excited through discrete transverse connections. With a reduction in the number of transverse connections, longitudinal conduction in the stimulated strand was still fast, but the unstimulated strand was activated almost simultaneously along its entire length after a pronounced delay. Depolarizing current flowing through a number of transverse connections was required to bring the unstimulated strand to its threshold. Under these conditions, the fast action potential upstroke in the unstimulated strand was still dependent mainly on the sodium current, but the calcium current was pivotal in the stimulated strand to maintain the action potential plateau to allow sufficient time for the unstimulated parallel strand to be activated. In this sense, continuous longitudinal and discontinuous transverse propagation show diverging dependencies on the calcium and sodium currents.

## 3. Fibrosis as a Proarrhythmic Factor

Fibrosis can be an underlying pathogenic factor in both brady- and tachyarrhythmias. 

Bradyarrhythmias result from conduction block in the sinus node or atrioventricular node, which is often caused by fibrosis, as a result of ageing, infarction, or other degenerative tissue changes [18,19].

For tachyarrhythmias, the two main mechanisms are reentry and ectopy. In reentry, one or more waves are continually propagating through the tissue, either in a regular and organized way (e.g., atrial flutter), or in a more chaotic and irregular pattern (e.g., ventricular fibrillation). In ectopy, sites within the tissue behave abnormally and generate action potentials, which then propagate to the rest of the tissue. The vulnerability to both ectopy and reentry can be increased by fibrosis. 

## 4. Fibrosis and Ectopy

Ectopy is thought to be caused by cellular abnormalities. In experiments with isolated myocytes, the corresponding phenomena are thought to be automaticity based on a gradual diastolic depolarization, and early or late afterdepolarizations resulting from abnormal calcium handling triggered by a preceding action potential (triggered activity). In isolated myocytes, these proarrhythmic events can reach the excitation threshold and thereby result in an action potential. However, exactly how and when these cellular proarrhythmic events lead to a propagating action potential in the intact tissue is still poorly understood. A depolarizing current during diastole, either by gradual spontaneous depolarization, early afterdepolarizations (caused by the reopening of the membrane calcium channel during repolarization), or late afterdepolarization (caused by a depolarizing current generated by the sodium–calcium exchanger during the removal of excess cytoplasmic calcium), can occur in an abnormal myocyte. However, in well-coupled tissue, a depolarizing current generated during diastole in a single cell would dissipate by leaking away to neighboring myocytes without reaching the action potential threshold [20]. A propagating action potential can only occur when cellular proarrhythmic events are synchronized among a sufficiently large cluster of adjoining myocytes. Strong electrical coupling would actually make the propagation of ectopic activity from a locus with abnormal myocytes less likely because the surrounding normal myocardium would effectively ‘silence’ the ectopic activity by ‘clamping’ the focus to its negative resting membrane potential. This apparent discrepancy was recognized in early research on pacemaking in the sinus node [21,22]. The sinus node is able to function as a pacemaker because pacemaker myocytes within the sinus node are poorly coupled, facilitating the ‘democratic synchronization’ of diastolic depolarization [23]. The pacemaker action potential is then able to propagate from the small sinus node to the much larger surrounding atrial myocardium more easily with a gradual increase in electrical coupling from the pacemaking area to the working myocardium [21]. In subsequent simulation studies, comparable phenomena were described for propagation from an ectopic focus to the surrounding myocardium, where anisotropy [24] or transverse resistive barriers [25] facilitated ectopic activity. Although not explicitly investigated in the context of ectopic activity, it is likely that increased fibrosis has effects similar to decreased gap junctional coupling: the corresponding loss of electrical connections would allow for the democratic synchronization of proarrhythmic events within a diseased tissue area and heterogeneity in coupling to the surrounding normal myocardium would facilitate the propagation of ectopic activity. In this way, fibrosis can reduce the ‘critical size’ required for an abnormal area to act as an ectopic focus, and thereby increase the likelihood of ectopic activity. 

An intriguing mechanism for the spatiotemporal synchronization of DADs has been described by Qu et al. In a mathematical model with APD variability due to the stochastic gating of ion channels, DADs synchronized over large areas in a pattern that showed pronounced beat-to-beat spatial variability [26]. In a later study with a similar model, the authors showed that the likelihood of this behavior was increased with decreasing gap junctional coupling and increasing tissue heterogeneity, both of which can be a consequence of fibrosis [22].

## 5. Fibrosis and Reentrant Activity

Compared to ectopic activity, the relation between fibrosis and reentrant activity has been investigated more extensively. The types of reentry thought to be most relevant for cardiac arrythmias are anatomic reentry, spiral wave reentry, and multiple wavelet reentry. These types are not only distinguished by different conduction patterns, but also have different relations to fibrosis. In anatomic reentry, an activation wavefront can travel around a circuit in one direction and re-excite previously excited tissue, leading to sustained reentrant movement of activation around an obstacle. To allow reentry to occur, the wavefront must continuously encounter tissue that has recovered from the previous excitation. A lower conduction velocity (CV) of the wave around the ring and a faster recovery time of the tissue (i.e., a shorter effective refractory period, ERP) increase the likelihood that anatomical reentry can occur. The wavelength (WL) of the reentrant wave, i.e., the product of CV and ERP, is equal to the minimal ring size able to sustain reentry [27]. If the ring size is larger, there will be an excitable gap, a delay between the recovery from a previous excitation and the start of the next. Anatomic reentry may explain why ventricular infarcts are associated with an increased vulnerability to reentrant ventricular tachycardias [3]. Fibrosis may precipitate anatomic reentry by forming a large obstacle at the center of the circuit, where dead myocytes have been replaced by fibrous tissue. In the infarct border zone, structural remodeling can cause slow conduction of the reentrant wave in the excitable tissue around this obstacle. Interestingly, de Bakker et al. have demonstrated that, even in the central infarct zone, thin strands of myocytes may still survive. These strands, surrounded by fibrous tissue, can sustain zig-zag conduction, allowing tortuous propagation pathways in the infarct zone that can form an integral part of a reentrant circuit [28,29].

Another type of reentrant wave is based on the general mathematical theory of spiral waves in excitable media [30]. This theory predicts the occurrence of rotating excitation waves with a rotor-shaped pattern that may be sustained depending on the curvature at the core of the wavefront. The stability of such a functional reentry wave does not have the simple relation to the ERP and CV as an anatomic reentry circuit and may occur in much smaller tissue areas. At a distance to the core of the rotor, the wavefront may encounter slowly conducting or refractory tissue and break up, causing more chaotic fibrillatory conduction in the rest of the tissue [31]. In tissue culture models, obstacles tend to destabilize rotors. In a nearly pure monolayer of neonatal myocytes, stable reentry of a single spiral wave can occur. However, with the addition of a larger proportion of fibroblasts to the monolayer, propagation patterns become more complex, forming a pattern more reminiscent of multiple wavelet reentry [32]. A single fibrotic obstacle can lead to ‘rotor pinning’, anchoring the spiral wave. However, in mathematical models, the effect of fibrosis on the dynamics of spiral wave reentry is highly dependent on the exact assumptions by which fibrosis is modelled [33]. This complex relation was underscored by Vandersickel et al., who showed that without fibrotic scars, the rotors were stable at the site of their initiation. In the presence of a scar, rotors were eventually dynamically anchored from large distances by the fibrotic scar via a process of dynamical reorganization of the excitation pattern [34].

In the description of multiple wavelet reentry, Moe and Abildskov used a computer model to show that reentrant waves can wander around refractory tissue in a seemingly chaotic pattern [35]. Individual wavelets may extinguish, split up, or fuse, but as long as a certain number of wavelets remains present, multiple wavelet reentry will be sustained. Similar to anatomic reentry, a shortened ERP, reduced CV, and larger size of the substrate also increase the stability of multiple wavelet reentry, because all three factors will increase the number of wavelets that can propagate simultaneously. In addition, heterogeneity in ERP and CV favors multiple wavelet reentry. Wavefronts move around refractory and slowly conducting tissue, increasing the tortuosity of conduction paths and the number of wavelets that can be present simultaneously within a certain area of tissue. Such heterogeneity in conduction may occur at a very small scale, as a result of stronger longitudinal versus weaker transverse gap junctional coupling, and the presence of endomysial and perimysial fibrous tissue. Spach and co-workers have argued that this leads to ‘nonuniform anisotropy’ in conduction, which may allow ‘micro’-reentry to occur in very small areas of tissue [36].

In this context, an interesting example of non-uniform anisotropy has been reported for the aging atrium. In a study comparing young and old dogs, Koura et al. showed that the degree of fibrosis and fat infiltration between strands of myocytes increased with age, while gap junction proteins became increasingly concentrated at end-to-end connections (Figure 3) [7]. In the terminal crest, a tissue area with a strong preferential fiber orientation, high-resolution optical mapping in old dogs revealed a pronounced anisotropy of conduction with extremely slow transverse conduction, causing a zig-zag conduction pattern. It is conceivable that transverse dissociation of conduction between fibers is a general characteristic of anisotropic atrial areas during aging. 

An additional, somewhat complementary description was provided by Vigmond et al., characterized as ‘percolation’ of electrical propagation [37]. In densely fibrotic areas, where myocytes intermingle with fibrosis, source-to-sink mismatches at a microscopic scale leads to heterogeneous conduction favoring reentry.

Another form of heterogeneity that can contribute to arrhythmogenesis is alternans, a beat-to-beat fluctuation in action potential duration, conduction velocity, and/or calcium transients. In particular, spatially discordant alternans, i.e., when adjacent regions alternate out of phase, can predispose to reentry [38]. In computer models including myocyte-to-fibroblast coupling and in co- cultures of neonatal myocytes and fibroblasts (see Section 8.4), spatially discordant alternans were more readily observed [39,40]. However, another mathematical model demonstrated that structural heterogeneity caused by simulated fibrosis was sufficient to provoke spatially discordant alternans [41]. The contribution of fibrosis to discordant alternans has been experimentally supported in the ventricles of aged rats during glycolytic inhibition [42] and in human ventricles with non-ischemic cardiomyopathy [43].

## 6. Association of Fibrosis with Arrhythmogenesis in Patients and Animal Models 

**Figure 5:**
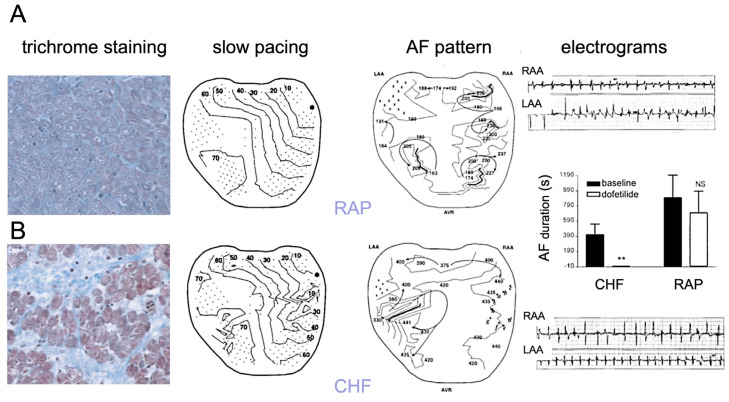
Fibrosis and conduction in dog models of AF. (**A**) In the model with 6–8 weeks of rapid atrial pacing (RAP) mimicking AF, no increase in fibrosis was detected (trichrome staining, left panel), and conduction during pacing was relatively homogeneous (asterisk indicates pacing site). (**B**) By contrast, the model subjected to 5–6 weeks of rapid ventricular pacing, leading to congestive heart failure (CHF), showed pronounced left atrial fibrosis and conduction heterogeneity during pacing. However, during AF, propagation patterns in the RAP models were more complex, with a higher degree of electrogram fractionation, compared to the patterns in the CHF model. The AF duration in the CHF model was also decreased by a larger degree compared to the RAP model (middle panel on the right), adapted from [52,55].

The importance of fibrosis as a pivotal proarrhythmic factor is widely recognized, but the exact relation to conduction disturbances is complex [44]. Fibrosis can play a role in the arrhythmias linked to the cardiac manifestations of inherited disorders, such as the replacement fibrosis seen in patients with Fabry disease [45]. Intriguingly, fibrosis has also been observed with some channelopathies in patients and animal models, most notably in the decreased expression of or mutations in the cardiac sodium channel SCN5A in Lenegre’s disease and Brugada syndrome, respectively [46,47], indicating a poorly understood relation between abnormal myocyte electrophysiology and alterations in tissue structure. However, much more commonly, fibrosis occurs as a consequence of acquired heart disease. In the ventricles with ischemic, dilated, or hypertrophic cardiomyopathy, fibrosis is associated with VT and VF [3,48,49]. The life-threatening decrease in pump function during ventricular arrhythmias makes studying the correlation between fibrosis and conduction patterns in vivo challenging. By contrast, this correlation can be studied more easily in the atrium during AF. Atrial arrhythmias are not directly life-threatening, affording more opportunity for detailed mapping studies, and the atria seem to have a stronger tendency to develop fibrosis. A mouse model overexpressing TGFβ1 showed atrial fibrosis in the atria, but not in the ventricles, and an increased inducibility and stability of AF, indicating that atrial fibrosis by itself is sufficient to cause AF [50]. The proarrhythmic mechanisms in this model may include both reentry [50] and ectopy [51]. In the field of AF research, Nattel and co-workers have extensively investigated and compared two canine models [52]. In a model of lone AF (i.e., AF without preexisting structural heart disease), the atria were rapidly paced to mimic AF (RAP, rapid atrial pacing), while the ventricular rate was controlled. After 6–8 weeks, the APD and ERP had shortened, but no increase in fibrosis was apparent. By contrast, in a canine model of congestive heart failure caused by five weeks of rapid ventricular pacing, the left atrium in particular displayed large areas of fibrous tissue deposition, suggestive of reparative fibrosis to replace dead myocytes. Left ventricular fibrosis also increased in this model, but to a far smaller extent than left atrial fibrosis [53], underscoring the stronger tendency of the atria to develop fibrosis. Indeed, a phase with marked apoptosis and necrosis of atrial myocytes had occurred within two days of the onset of ventricular pacing, at an early stage in the gradual development of CHF in this model [54]. Conduction heterogeneity, a measure for activation delays between neighboring recording electrodes, was increased in the left atrium of the CHF model, but not in the RAP model [52]. However, during AF, fibrillation patterns were less complex in the CHF model and pharmacological cardioversion with dofetilide was more effective compared to the RAP model (Figure 5) [55]. In the canine CHF model, both atrial fibrosis and atrial conduction abnormalities can be inhibited by enalapril, inhibiting the angiotensin converting enzyme [56], and by the anti-fibrotic drug pirfenidone [57]. In addition, during recovery from CHF after the cessation of rapid ventricular pacing, cardiac chamber dimensions and function normalizes, but atrial fibrosis, conduction heterogeneity, and AF vulnerability remained elevated, strengthening the evidence that atrial fibrosis is a pivotal proarrhythmic factor in this model [58].

In the dog RAP model, ventricular rate was controlled by His bundle ablation and a ventricular pacemaker. Studies in goat and sheep models have shown that atrial structural remodeling, including fibrosis, to a large extent depends on a high ventricular response during RAP [59,60]. In the goat model, RAP leads to rapid electrical remodeling (APD/ERP shortening) within the first 1–2 days, and much slower structural remodeling over a time course of months. The latter is associated with an increase in the complexity of fibrillation patterns, leading to more numerous, smaller waves, and a complete loss of efficacy of antiarrhythmic drugs [61]. In this model, the total relative area occupied by fibrous tissue was unaltered after 6 months of AF, but there was an increase in endomysial fibrosis, quantified as myocyte-to-myocyte distance in the outer 0.5–1 mm across the atrial walls [62]. This layer corresponds to the thin epicardial wall overlying the endocardial trabecular network and optical mapping recordings showed a concomitant impairment of transverse propagation in this layer. We have hypothesized that epicardial endomysial fibrosis produces a loss of the synchronizing effect of the epicardial layer, leading to dissociation of the epicardial layer and the underlying bundle network and a more three-dimensional fibrillation pattern [63]. This idea, which we have subsequently tested using a detailed atrial computer model [64], provides an example of a large effect on conduction caused by a small increase in fibrosis in a small portion of the tissue.

The clinical relationship between fibrosis and AF is less straightforward because of the presence of underlying risk factors in many patients. Anne et al. reported that mitral valve disease, a well-recognized risk factor for AF, leads to increased atrial fibrosis, but that AF itself was not associated with increased atrial fibrosis [65]. By contrast, Platonov et al. reported increased fibrosis in patients with AF, and that fibrosis was more pronounced in patients with permanent AF than in patients with paroxysmal AF [66].

## 7. Direct Correlation of Conduction Abnormalities to Fibrosis

**Figure 6:**
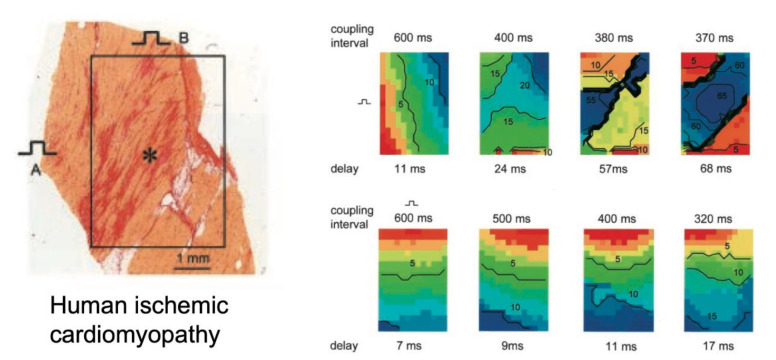
Impact of fibrotic strands in ventricular cardiomyopathy. In an explanted epicardial tissue slice with long strands of fibrosis, conduction patterns during extrastimulation with progressively shorter intervals were recorded for two different pacing sites (top and bottom row). During pacing at site B, the longitudinal wavefront showed a modest decrease in conduction velocity (bottom row), while at pacing site A, the propagating wavefront showed pronounced transverse conduction block, adapted from [68].

Despite the strong association between fibrosis and the proarrhythmic conduction disturbances mentioned above, and the appealing fact that this association makes intuitive sense, direct demonstrations for a causal relation are surprisingly rare. A first reason is that a direct one-to-one correlation of fibrous tissue and alterations in conduction is very difficult. At the highest resolution, both direct contact electrical mapping and optical mapping can in practice reveal disrupted conduction on a millimeter scale. These disruptions may not be noticeable during slow rhythms, when the safety factor of conduction is high due to complete recovery of the sodium current, even in structurally abnormal tissue. They may only become apparent during extrastimulation with short coupling intervals [11,67] or during fibrillatory conduction [61], when incomplete recovery of the sodium current increases source-to-sink mismatches. By contrast, the underlying fibrotic structures and their visualization require careful tissue analysis at a micrometer scale. Conventional histological techniques used for these purposes entail tissue sample, fixation, and sectioning artefacts that complicate direct matching to mapping data from the same region, allowing only for correlation to average tissue characteristics. A rare and interesting example of this approach was presented by Kawara et al. for ventricular epicardial tissue slices from human hearts with cardiomyopathy [68]. Extrastimulation caused a marked increase in activation delay during transverse propagation in zones with dense, patchy fibrosis with long fibrotic strands (Figure 6). Dense, diffuse fibrosis with short fibrotic strands did not show a similar effect on conduction, despite a similar amount of total fibrosis. In the same tradition, Krul et al. showed that in the left atrium, thick fibrotic strands were associated with faster longitudinal conduction and unaltered transverse conduction during slow pacing, but activation times were increased during extrastimulation compared to tissue with thinner fibrotic strands [69].

Some successful attempts have been made to resolve fibrous tissue distribution using other techniques. For example, Hansen et al. used magnetic resonance imaging of atrial tissue with late gadolinium enhancement to detect fibrosis at a pixel resolution of 80 mm^3^. They observed that, in human right atria perfused with pinacidil (shortening the APD), AF was driven by intramural reentry anchored to atrial bundles insulated by fibrosis [70]. In patients, late gadolinium enhancement imaging has been used in recent years to detect fibrosis, although at much lower resolutions [71,72]. This method has been well validated histologically for large infarct scars in the thick ventricular wall, but a direct histological validation of delayed enhancement imaging of the much thinner atrial wall is limited [73]. Therefore, the exact type and distribution of fibrous tissue (i.e., endomysial/perimysial fibrosis vs. replacement fibrosis) detected using this technique remains unclear. Nevertheless, areas with delayed enhancement have been correlated to regions with low-voltage electrograms [74,75], and can be predictive of clinical outcomes. Most notably, patient-specific mathematical models incorporating information from delayed enhancement imaging have been successfully used to guide ablation strategies, both in AF [76] and VT [77].

The implementation of the effects of fibrosis in computer models critically depends on the underlying assumptions about the distribution of electrical connections in healthy tissue, and on how these connection patterns are affected by fibrosis [78]. For detailed current models, even with a high degree of simulated fibrosis, fibrillation patterns are still relatively simple [64] compared to patterns observed in epicardial mapping studies in vivo [79,80]. Diffusion-tensor imaging and detailed histological reconstructions have elucidated the orientation of myocardial fibers [81,82].

Most models assume a continuous, homogenous distribution of high longitudinal and lower transverse connectivity. However, we have limited understanding of the actual distribution of electrical connections between these fibers and their regional variability, particularly regarding the role of discrete, sparse transverse connections and their sensitivity to disruption by fibrosis.

## 8. Confounding Factors in Determining the Impact of Fibrosis on Propagation

Another reason complicating investigation of the effects of fibrosis is that, under pathological conditions, fibrosis often occurs in combination with other factors that may influence conduction, acting as confounding factors in understanding the effects of fibrosis. For example, fibrosis often coincides with fatty infiltration, myocyte hypertrophy, altered gap junction distribution, increased fibroblast density, and alterations in myocyte electrophysiology.

### 8.1. Fatty Infiltration

In some diseases, an increased presence of fatty infiltrates occurs in conjunction with fibrosis, as demonstrated, for example, in the atria of a sheep model of obesity [83] and in the right ventricle in arrhythmogenic right ventricular cardiomyopathy [84]. In principle, fatty infiltrates may have a similar effect on propagation as fibrosis, with adipocytes forming non-conducting barriers in between strands of myocytes, but the direct relation between fatty infiltrates and alterations in propagation has not been studied as extensively [85].

### 8.2. Myocyte Hypertrophy

Myocyte hypertrophy has been observed in animal models of AF [86], atrial dilatation [87,88], and CHF [52] in the atria and in the ventricles during heart failure [89]. The contribution of cellular hypertrophy to alterations in conduction is difficult to ascertain. From cable theory, it is expected that an increase in myocyte width would increase longitudinal conduction velocity. Indeed, in a rabbit model of heart failure, myocyte hypertrophy was associated with an increase in macroscopic conduction velocity [89]. By contrast, using a detailed atrial mathematical model that took the nonuniform distribution of gap junction around myocytes into account, Spach et al. [90,91] calculated that an increase in cell size by itself would lead to more pronounced propagation delays between myocytes during transverse propagation. In addition to the larger overall size of dilated atria, this finding may explain how myocyte hypertrophy in the absence of increased fibrosis can also cause conduction disturbances, as has been reported for a goat model of gradual, chronic atrial dilatation caused by AV block [88].

### 8.3. Connexin Expression

In cardiomyopathy, ventricular myocytes show increased ‘lateralization’ of connexin43, the main gap junction protein (reviewed extensively in [6]). It is still under debate whether an increase in Cx43 present at lateral myocyte borders increases transverse conduction [92], presenting a compensatory mechanism [93], or whether these subunit form hemichannels that can be non-functional or even contribute to electrical instability [94]. For the atria, where myocytes normally express both connexin43 and connexin40, the contribution of altered connexin expression is even less clear [6]. During AF in the goat model, a heterogenous loss of Cx40 was observed, while in AF patients, Cx40 was increased, and both atrial connexins were lateralized [95]. It is unclear whether a decrease in Cx40 expression will decrease conduction velocity, as observed in transgenic mice lacking Cx40 [96], or increase conduction velocity, as has been observed in human atria [97,98].

### 8.4. Fibroblast Density

The contribution of electrical coupling between myocytes and fibroblasts to abnormal propagation in the adult myocardium is still not well understood. Most evidence stems from co-cultures of neonatal rat myocytes and fibroblasts, where these cell types may more readily form gap junctions [99]. Such studies have demonstrated that, in principle, fibroblasts can bridge a discontinuity between myocytes and thus mediate propagation over a distance of 0.3 mm [100]. In the adult heart, direct contact between the fibroblast and myocytes has been demonstrated in the sinoatrial node, where myocytes intermingle with fibrous tissue, possibly affecting pacemaker activity [101]. In the ventricular scar border zones, the most direct evidence to date for electrical coupling between myocytes and non-excitable non-myocytes (most likely (myo)fibroblasts) was provided by Quinn et al. using an optogenetic approach [102]. This finding was confirmed in the border zone of ventricular infarcts in mice [103]. Depending on the underlying assumptions, electrical coupling between fibroblasts and myocytes can have a pronounced impact on conduction in mathematical models [104], but the role of this phenomenon in vivo remains poorly understood.

### 8.5. Electrical Remodeling

In many cases, structural remodeling coincides with changes in cellular electrophysiology. In ventricular cardiomyopathy, reactive fibrosis coincides with APD prolongation and altered calcium handling, which increase the likelihood of proarrhythmic EADs and DADs. The synchronization and propagation of these events can be enhanced by fibrosis, as detailed above. In AF, structural remodeling takes place on a background of electrical remodeling, shortening the APD. In a seminal study, Schuessler et al. demonstrated that APD shortening by the application of acetylcholine (ACh) can change the nature of AF from a pattern resembling multiple wavelet reentry at low concentration to one coalescing around a functional line of block that shortens with higher concentrations, increasingly resembling spiral wave reentry [105]. As detailed above, these different types of reentry may interact in different ways with fibrotic obstacles.

Another factor that is thought to be proarrhythmic is regional heterogeneity in APD. Modeling studies have confirmed the effect of APD dispersion on arrhythmogenesis [106]. Electrotonic interactions govern both the propagation of activation and repolarization wavefronts [107]. In principle, strong electrical coupling would produce a less steep gradient in repolarization and, conversely, a reduction in electrical coupling caused by fibrosis may allow steeper gradients to occur. However, the extent to which this effect contributes to the initiation and maintenance of reentrant conduction remains poorly understood. 

## 9. Histological Quantification of Fibrosis

As detailed above, various types of fibrosis can contribute to conduction disturbances in different ways. To specifically quantify the types of fibrosis that can affect conduction, other types of fibrous tissue have to be excluded from the analysis. Thus, perivascular, epicardial, and endocardial fibrous tissue should not be photographed, or should be erased from photographs before quantification. In the thin, trabeculated parts of the atria, tissue slices include invaginations of the endocardial layer in between trabeculae, sometimes complicating recognition, and exclude endocardial fibrous tissue. 

**Figure 7:**
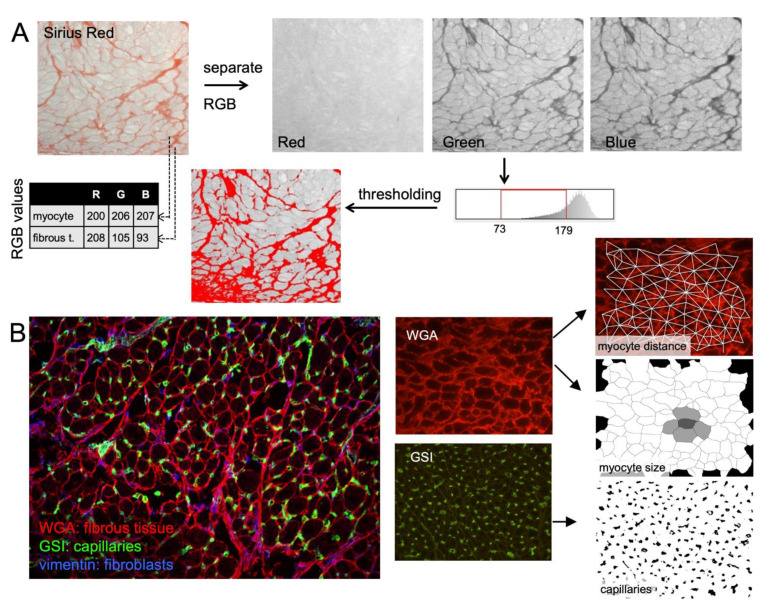
Histological quantification of fibrosis. (**A**) Sirius Red staining of goat atrial tissue: fibrous tissue is stained red, myocytes light green. Analysis of local red–green–blue (RGB) values illustrate significant overlap: the red value of myocytes is similar to that of fibrous tissue, although the green value of fibrous tissue is lower, allowing separation based on a user-defined threshold of the green image. However, endomysial fibrous tissue is poorly resolved, and the separation is sensitive to inhomogeneous illumination (relatively dark area at bottom left). (**B**) Fluorescent labelling of goat atrial tissue using wheat germ agglutinin (WGA, extracellular matrix in red), *Griffonia Simplicfolia* I (GSI, endothelial cells in green) and anti-vimentin antibody (fibroblasts in blue). Color-separated images allow automated thresholding, followed by quantification of endomysial fibrosis (myocyte-to-myocyte distance), overall fibrosis (percentage of red pixels), and myocyte size (short axes of dark areas). The structure of the extracellular matrix can then be correlated to other markers, e.g., the distribution of capillaries. Adapted from [108].

The most commonly used staining methods for the quantification of fibrosis are Sirius Red (myocytes yellow-green, fibrous tissue red) and Masson’s trichrome (myocyte red, fibrous tissue blue). Most studies differentiate between myocytes and fibrous tissue by setting a color threshold to recognize fibrous tissue, in order to determine the ratio between the surface occupied by fibrous tissue to myocytes. However, the RGB (red–green –blue) values of myocytes and fibrous tissue can show considerable overlap. Thus, there is no objective threshold to perfectly differentiate myocytes from fibrous tissue, and this method is sensitive to the effects of inhomogeneities in illumination. Setting the threshold is often performed manually for each individual photograph, potentially introducing observer bias, and making blinded analysis essential (Figure 7). 

The resulting measure for fibrosis, often termed ‘interstitial fibrosis’, is a combination of endomysial, perimysial, and replacement fibrosis. In addition, both Sirius Red and trichrome staining show clear staining of larger areas of fibrous tissue, but are not particularly sensitive to endomysial fibrous tissue, which also affects conduction. As a result of these uncertainties, studies differ widely in the amount of fibrous tissue observed in healthy control animals, e.g., from 1% [52], 5% [109] to 8% [110] in dog atria and from 5% [83], 7% [60] to 14% [111] in sheep atria. While this variation may reflect interspecies differences, considerable variation is also present in different studies on the same species. 

To remedy some of these problems, we have recently developed a staining method and analysis algorithms for the fast, unbiased quantification of fibrosis and other tissue structure parameters [108]. Using wheat germ agglutinin (WGA) to stain the extracellular matrix, endomysial fibrosis, overall fibrosis, and myocyte hypertrophy can be quantified automatically. This method also allows combination with antibodies to label specific structures (e.g., fibroblasts, capillaries, macrophages) and to determine their clustering and spatial relation to fibrous tissue. For automated, large volume quantification of structural characteristics, this and other similar methods may be convenient, but several challenges remain in the elucidation of the role of fibrosis in altered cardiac propagation and arrhythmogenesis. 

## 10. Conclusions

Fibrosis has long been recognized as a pivotal factor in arrhythmogenesis. A strong association has been observed in various pathologies in many different patient populations and has been confirmed in animal models of cardiac disease. Nevertheless, several intriguing questions remain concerning the exact relation of fibrosis and substrate for arrhythmias. Addressing these questions requires a fuller understanding of the nature and distribution of electrical connections between myocytes in the normal heart, and the disruption of these connections by various distinctly different types of fibrous tissue. Finding answers to these open questions is likely to involve a multidisciplinary approach with detailed reconstruction of tissue structures and the correlation to propagation patterns observed in mapping studies in conjunction with mathematical models.

## Data Availability

Not applicable.

## References

[1] Weber K.T., Pick R., Jalil J.E., Janicki J.S., Carroll E.P. (1989). Patterns of myocardial fibrosis. J. Mol. Cell. Cardiol..

[2] Weber K.T., Sun Y., Tyagi S.C., Cleutjens J.P. (1994). Collagen network of the myocardium: Function, structural remodeling and regulatory mechanisms. J. Mol. Cell. Cardiol..

[3] Frangogiannis N.G. (2021). Cardiac fibrosis. Cardiovasc. Res..

[4] Dai Z., Aoki T., Fukumoto Y., Shimokawa H. (2012). Coronary perivascular fibrosis is associated with impairment of coronary blood flow in patients with non-ischemic heart failure. J. Cardiol..

[5] Marques F.Z., Chu P.Y., Ziemann M., Kaspi A., Kiriazis H., Du X.J., El-Osta A., Kaye D.M. (2018). Age-Related Differential Structural and Transcriptomic Responses in the Hypertensive Heart. Front. Physiol..

[6] Dhein S., Salameh A. (2021). Remodeling of Cardiac Gap Junctional Cell-Cell Coupling. Cells.

[7] Koura T., Hara M., Takeuchi S., Ota K., Okada Y., Miyoshi S., Watanabe A., Shiraiwa K., Mitamura H., Kodama I. (2002). Anisotropic conduction properties in canine atria analyzed by high-resolution optical mapping: Preferential direction of conduction block changes from longitudinal to transverse with increasing age. Circulation.

[8] Dolber P.C., Spach M.S. (1989). Structure of canine Bachmann’s bundle related to propagation of excitation. Am. J. Physiol..

[9] Clerc L. (1976). Directional differences of impulse spread in trabecular muscle from mammalian heart. J. Physiol..

[10] Hansson A., Holm M., Blomstrom P., Johansson R., Luhrs C., Brandt J., Olsson S.B. (1998). Right atrial free wall conduction velocity and degree of anisotropy in patients with stable sinus rhythm studied during open heart surgery. Eur. Heart J..

[11] Spach M.S., Dolber P.C., Heidlage J.F. (1990). Properties of discontinuous anisotropic propagation at a microscopic level. Ann. N. Y. Acad. Sci..

[12] Spach M.S., Dolber P.C. (1986). Relating extracellular potentials and their derivatives to anisotropic propagation at a microscopic level in human cardiac muscle. Evidence for electrical uncoupling of side-to-side fiber connections with increasing age. Circ. Res..

[13] Spach M.S., Heidlage J.F. (1995). The stochastic nature of cardiac propagation at a microscopic level. Electrical description of myocardial architecture and its application to conduction. Circ. Res..

[14] Shaw R.M., Rudy Y. (1997). Ionic Mechanisms of Propagation in Cardiac Tissue: Roles of the Sodium and L-type Calcium Currents During Reduced Excitability and Decreased Gap Junction Coupling. Circ. Res..

[15] Joyner R.W., Kumar R., Wilders R., Jongsma H.J., Verheijck E.E., Golod D.A., van Ginneken A.C., Wagner M.B., Goolsby W.N. (1996). Modulating L-type calcium current affects discontinuous cardiac action potential conduction. Biophys. J..

[16] Rohr S., Kucera J.P., Fast V.G., Kléber A.G. (1997). Paradoxical improvement of impulse conduction in cardiac tissue by partial cellular uncoupling. Science.

[17] Zhao J., Schotten U., Smaill B., Verheule S. (2018). Loss of Side-to-Side Connections Affects the Relative Contributions of the Sodium and Calcium Current to Transverse Propagation Between Strands of Atrial Myocytes. Front. Physiol..

[18] Choudhury M., Boyett M.R., Morris G.M. (2015). Biology of the Sinus Node and its Disease. Arrhythm. Electrophysiol. Rev..

[19] Waller B.F., Gering L.E., Branyas N.A., Slack J.D. (1993). Anatomy, histology, and pathology of the cardiac conduction system—Part V. Clin. Cardiol..

[20] Xie Y., Sato D., Garfinkel A., Qu Z., Weiss J.N. (2010). So little source, so much sink: Requirements for afterdepolarizations to propagate in tissue. Biophys. J..

[21] Joyner R.W., van Capelle F.J. (1986). Propagation through electrically coupled cells. How a small SA node drives a large atrium. Biophys. J..

[22] Weiss J.N., Qu Z. (2020). The Sinus Node: Still Mysterious After All These Years. JACC Clin. Electrophysiol..

[23] Jalife J., Michaels D.C., Delmar M. (1988). Mechanisms of pacemaker synchronization in the sinus node. Prog Clin. Biol. Res..

[24] Wilders R., Wagner M.B., Golod D.A., Kumar R., Wang Y.G., Goolsby W.N., Joyner R.W., Jongsma H.J. (2000). Effects of anisotropy on the development of cardiac arrhythmias associated with focal activity. Pflügers Arch. Eur. J. Physiol..

[25] Wang Y.G., Kumar R., Wagner M.B., Wilders R., Golod D.A., Goolsby W.N., Joyner R.W. (2000). Electrical interactions between a real ventricular cell and an anisotropic two-dimensional sheet of model cells. Am. J. Physiol. Heart Circ. Physiol..

[26] Sato D., Xie L.-H., Sovari A.A., Tran D.X., Morita N., Xie F., Karagueuzian H., Garfinkel A., Weiss J.N., Qu Z. (2009). Synchronization of chaotic early afterdepolarizations in the genesis of cardiac arrhythmias. Proc. Natl. Acad. Sci. USA.

[27] Wiener N., Rosenblueth A. (1946). The mathematical formulation of the problem of conduction of impulses in a network of connected excitable elements, specifically in cardiac muscle. Arch. Inst. Cardiol. Mex..

[28] de Bakker J., Coronel R., Tasseron S., Wilde A., Opthof T., Janse M., van Capelle F., Becker A., Jambroes G. (1990). Ventricular tachycardia in the infarcted, Langendorff-perfused human heart: Role of the arrangement of surviving cardiac fibers. J. Am. Coll. Cardiol..

[29] de Bakker J., van Capelle F., Janse M., Tasseron S., Vermeulen J., de Jonge N., Lahpor J. (1993). Slow conduction in the infarcted human heart. ‘Zigzag’ course of activation. Circulation.

[30] Pertsov A.M., Davidenko J.M., Salomonsz R., Baxter W.T., Jalife J. (1993). Spiral waves of excitation underlie reentrant activity in isolated cardiac muscle. Circ. Res..

[31] Kalifa J., Tanaka K., Zaitsev A.V., Warren M., Vaidyanathan R., Auerbach D., Pandit S., Vikstrom K.L., Ploutz-Snyder R., Talkachou A. (2006). Mechanisms of wave fractionation at boundaries of high-frequency excitation in the posterior left atrium of the isolated sheep heart during atrial fibrillation. Circulation.

[32] Zlochiver S., Muñoz V., Vikstrom K.L., Taffet S.M., Berenfeld O., Jalife J. (2008). Electrotonic myofibroblast-to-myocyte coupling increases propensity to reentrant arrhythmias in two-dimensional cardiac monolayers. Biophys. J..

[33] Roney C.H., Bayer J.D., Zahid S., Meo M., Boyle P.M., Trayanova N.A., Haissaguerre M., Dubois R., Cochet H., Vigmond E.J. (2016). Modelling methodology of atrial fibrosis affects rotor dynamics and electrograms. Europace.

[34] Vandersickel N., Watanabe M., Tao Q., Fostier J., Zeppenfeld K., Panfilov A.V. (2018). Dynamical anchoring of distant arrhythmia sources by fibrotic regions via restructuring of the activation pattern. PLoS Comput. Biol..

[35] Moe G.K., Abildskov J.A. (1959). Atrial fibrillation as a self-sustaining arrhythmia independent of focal discharge. Am. Heart J..

[36] Spach M., Boineau J. (1997). Microfibrosis produces electrical load variations due to loss of side-to-side cell connections: A major mechanism of structural heart disease arrhythmias. Pacing Clin. Electrophysiol..

[37] Vigmond E., Pashaei A., Amraoui S., Cochet H., Hassaguerre M. (2016). Percolation as a mechanism to explain atrial fractionated electrograms and reentry in a fibrosis model based on imaging data. Heart Rhythm.

[38] Weiss J.N., Karma A., Shiferaw Y., Chen P.-S., Garfinkel A., Qu Z. (2006). From pulsus to pulseless: The saga of cardiac alternans. Circ. Res..

[39] Xie Y., Garfinkel A., Weiss J.N., Qu Z. (2009). Cardiac alternans induced by fibroblast-myocyte coupling: Mechanistic insights from computational models. Am. J. Physiol. Heart Circ. Physiol..

[40] Majumder R., Engels M.C., de Vries A.A., Panfilov A.V., Pijnappels D.A. (2016). Islands of spatially discordant APD alternans underlie arrhythmogenesis by promoting electrotonic dyssynchrony in models of fibrotic rat ventricular myocardium. Sci. Rep..

[41] Engelman Z.J., Trew M.L., Smaill B.H. (2010). Structural heterogeneity alone is a sufficient substrate for dynamic instability and altered restitution. Circ. Arrhythm. Electrophysiol..

[42] Morita N., Lee J.H., Bapat A., Fishbein M.C., Mandel W.J., Chen P.S., Weiss J.N., Karagueuzian H.S. (2011). Glycolytic inhibition causes spontaneous ventricular fibrillation in aged hearts. Am. J. Physiol. Heart Circ. Physiol..

[43] Glukhov A.V., Fedorov V.V., Kalish P.W., Ravikumar V.K., Lou Q., Janks D., Schuessler R.B., Moazami N., Efimov I.R. (2012). Conduction remodeling in human end-stage nonischemic left ventricular cardiomyopathy. Circulation.

[44] Burstein B., Nattel S. (2008). Atrial fibrosis: Mechanisms and clinical relevance in atrial fibrillation. J. Am. Coll. Cardiol..

[45] Seydelmann N., Wanner C., Stork S., Ertl G., Weidemann F. (2015). Fabry disease and the heart. Best Pract. Res. Clin. Endocrinol. Metab..

[46] Royer A., van Veen T.A., Le Bouter S., Marionneau C., Griol-Charhbili V., Leoni A.L., Steenman M., van Rijen H.V., Demolombe S., Goddard C.A. (2005). Mouse model of SCN5A-linked hereditary Lenegre’s disease: Age-related conduction slowing and myocardial fibrosis. Circulation.

[47] Blok M., Boukens B.J. (2020). Mechanisms of Arrhythmias in the Brugada Syndrome. Int. J. Mol. Sci..

[48] Mc L.A., Ellims A.H., Prabhu S., Voskoboinik A., Iles L.M., Hare J.L., Kaye D.M., Macciocca I., Mariani J.A., Kalman J.M. (2016). Diffuse Ventricular Fibrosis on Cardiac Magnetic Resonance Imaging Associates With Ventricular Tachycardia in Patients With Hypertrophic Cardiomyopathy. J. Cardiovasc. Electrophysiol..

[49] Disertori M., Mase M., Ravelli F. (2017). Myocardial fibrosis predicts ventricular tachyarrhythmias. Trends Cardiovasc. Med..

[50] Verheule S., Sato T., Everett T., Engle S.K., Otten D., Rubart-von der Lohe M., Nakajima H.O., Nakajima H., Field L.J., Olgin J.E. (2004). Increased vulnerability to atrial fibrillation in transgenic mice with selective atrial fibrosis caused by overexpression of TGF-beta1. Circ. Res..

[51] Choi E.K., Chang P.C., Lee Y.S., Lin S.F., Zhu W., Maruyama M., Fishbein M.C., Chen Z., Rubart-von der Lohe M., Field L.J. (2012). Triggered firing and atrial fibrillation in transgenic mice with selective atrial fibrosis induced by overexpression of TGF-beta1. Circ. J..

[52] Li D., Fareh S., Leung T., Nattel S. (1999). Promotion of atrial fibrillation by heart failure in dogs: Atrial remodeling of a different sort. Circulation.

[53] Hanna N., Cardin S., Leung T.K., Nattel S. (2004). Differences in atrial versus ventricular remodeling in dogs with ventricular tachypacing-induced congestive heart failure. Cardiovasc. Res..

[54] Cardin S., Li D., Thorin-Trescases N., Leung T.K., Thorin E., Nattel S. (2003). Evolution of the atrial fibrillation substrate in experimental congestive heart failure: Angiotensin-dependent and -independent pathways. Cardiovasc. Res..

[55] Li D., Benardeau A., Nattel S. (2000). Contrasting efficacy of dofetilide in differing experimental models of atrial fibrillation. Circulation.

[56] Li D., Shinagawa K., Pang L., Leung T.K., Cardin S., Wang Z., Nattel S. (2001). Effects of angiotensin-converting enzyme inhibition on the development of the atrial fibrillation substrate in dogs with ventricular tachypacing-induced congestive heart failure. Circulation.

[57] Lee K.W., Everett T.H., Rahmutula D., Guerra J.M., Wilson E., Ding C., Olgin J.E. (2006). Pirfenidone prevents the development of a vulnerable substrate for atrial fibrillation in a canine model of heart failure. Circulation.

[58] Shinagawa K., Shi Y.-F., Tardif J.-C., Leung T.-K., Nattel S. (2002). Dynamic nature of atrial fibrillation substrate during development and reversal of heart failure in dogs. Circulation.

[59] Schoonderwoerd B.A., Ausma J., Crijns H.J., Van Veldhuisen D.J., Blaauw E.H., Van Gelder I.C. (2004). Atrial ultrastructural changes during experimental atrial tachycardia depend on high ventricular rate. J. Cardiovasc. Electrophysiol..

[60] Anne W., Willems R., Holemans P., Beckers F., Roskams T., Lenaerts I., Ector H., Heidbuchel H. (2007). Self-terminating AF depends on electrical remodeling while persistent AF depends on additional structural changes in a rapid atrially paced sheep model. J. Mol. Cell Cardiol..

[61] Verheule S., Tuyls E., van Hunnik A., Kuiper M., Schotten U., Allessie M. (2010). Fibrillatory conduction in the atrial free walls of goats in persistent and permanent atrial fibrillation. Circ. Arrhythmia Electrophysiol..

[62] Verheule S., Tuyls E., Gharaviri A., Hulsmans S., van Hunnik A., Kuiper M., Serroyen J., Zeemering S., Kuijpers N.H.L., Schotten U. (2013). Loss of Continuity in the Thin Epicardial Layer Due to Endomysial Fibrosis Increases the Complexity of Atrial Fibrillatory Conduction. Circ. Arrhythmia Electrophysiol..

[63] Verheule S., Eckstein J., Linz D., Maesen B., Bidar E., Gharaviri A., Schotten U. (2014). Role of endo-epicardial dissociation of electrical activity and transmural conduction in the development of persistent atrial fibrillation. Prog. Biophys. Mol. Biol..

[64] Gharaviri A., Bidar E., Potse M., Zeemering S., Verheule S., Pezzuto S., Krause R., Maessen J.G., Auricchio A., Schotten U. (2020). Epicardial Fibrosis Explains Increased Endo-Epicardial Dissociation and Epicardial Breakthroughs in Human Atrial Fibrillation. Front. Physiol..

[65] Anné W., Willems R., Roskams T., Sergeant P., Herijgers P., Holemans P., Ector H., Heidbüchel H. (2005). Matrix metalloproteinases and atrial remodeling in patients with mitral valve disease and atrial fibrillation. Cardiovasc. Res..

[66] Platonov P.G., Mitrofanova L.B., Orshanskaya V., Ho S.Y. (2011). Structural abnormalities in atrial walls are associated with presence and persistency of atrial fibrillation but not with age. J. Am. Coll. Cardiol..

[67] Verheule S., Wilson E., Everett T.t., Shanbhag S., Golden C., Olgin J. (2003). Alterations in atrial electrophysiology and tissue structure in a canine model of chronic atrial dilatation due to mitral regurgitation. Circulation.

[68] Kawara T., Derksen R., de Groot J.R., Coronel R., Tasseron S., Linnenbank A.C., Hauer R.N., Kirkels H., Janse M.J., de Bakker J.M. (2001). Activation delay after premature stimulation in chronically diseased human myocardium relates to the architecture of interstitial fibrosis. Circulation.

[69] Krul S.P.J., Berger W.R., Smit N.W., van Amersfoorth S.C.M., Driessen A.H.G., van Boven W.J., Fiolet J.W.T., van Ginneken A.C.G., van der Wal A.C., de Bakker J.M.T. (2015). Atrial fibrosis and conduction slowing in the left atrial appendage of patients undergoing thoracoscopic surgical pulmonary vein isolation for atrial fibrillation. Circ. Arrhythmia Electrophysiol..

[70] Hansen B.J., Zhao J., Csepe T.A., Moore B.T., Li N., Jayne L.A., Kalyanasundaram A., Lim P., Bratasz A., Powell K.A. (2015). Atrial fibrillation driven by micro-anatomic intramural re-entry revealed by simultaneous sub-epicardial and sub-endocardial optical mapping in explanted human hearts. Eur. Heart J..

[71] McGann C., Akoum N., Patel A., Kholmovski E., Revelo P., Damal K., Wilson B., Cates J., Harrison A., Ranjan R. (2014). Atrial fibrillation ablation outcome is predicted by left atrial remodeling on MRI. Circ. Arrhythmia Electrophysiol..

[72] Sohns C., Marrouche N.F. (2020). Atrial fibrillation and cardiac fibrosis. Eur. Heart J..

[73] Zghaib T., Keramati A., Chrispin J., Huang D., Balouch M.A., Ciuffo L., Berger R.D., Marine J.E., Ashikaga H., Calkins H. (2018). Multimodal Examination of Atrial Fibrillation Substrate: Correlation of Left Atrial Bipolar Voltage Using Multi-Electrode Fast Automated Mapping, Point-by-Point Mapping, and Magnetic Resonance Image Intensity Ratio. JACC Clin. Electrophysiol..

[74] Chen J., Arentz T., Cochet H., Muller-Edenborn B., Kim S., Moreno-Weidmann Z., Minners J., Kohl P., Lehrmann H., Allgeier J. (2019). Extent and spatial distribution of left atrial arrhythmogenic sites, late gadolinium enhancement at magnetic resonance imaging, and low-voltage areas in patients with persistent atrial fibrillation: Comparison of imaging vs. electrical parameters of fibrosis and arrhythmogenesis. Europace.

[75] Cochet H., Dubois R., Yamashita S., Al Jefairi N., Berte B., Sellal J.M., Hooks D., Frontera A., Amraoui S., Zemoura A. (2018). Relationship Between Fibrosis Detected on Late Gadolinium-Enhanced Cardiac Magnetic Resonance and Re-Entrant Activity Assessed With Electrocardiographic Imaging in Human Persistent Atrial Fibrillation. JACC Clin. Electrophysiol..

[76] Boyle P.M., Zghaib T., Zahid S., Ali R.L., Deng D., Franceschi W.H., Hakim J.B., Murphy M.J., Prakosa A., Zimmerman S.L. (2019). Computationally guided personalized targeted ablation of persistent atrial fibrillation. Nat. Biomed. Eng..

[77] Prakosa A., Arevalo H.J., Deng D., Boyle P.M., Nikolov P.P., Ashikaga H., Blauer J.J.E., Ghafoori E., Park C.J., Blake R.C. (2018). Personalized virtual-heart technology for guiding the ablation of infarct-related ventricular tachycardia. Nat. Biomed. Eng..

[78] Saliani A., Irakoze E., Jacquemet V. (2021). Simulation of diffuse and stringy fibrosis in a bilayer interconnected cable model of the left atrium. Europace.

[79] Allessie M.A., de Groot N.M., Houben R.P., Schotten U., Boersma E., Smeets J.L., Crijns H.J. (2010). Electropathological substrate of long-standing persistent atrial fibrillation in patients with structural heart disease: Longitudinal dissociation. Circ. Arrhythmia Electrophysiol..

[80] de Groot N.M., Houben R.P., Smeets J.L., Boersma E., Schotten U., Schalij M.J., Crijns H., Allessie M.A. (2010). Electropathological substrate of longstanding persistent atrial fibrillation in patients with structural heart disease: Epicardial breakthrough. Circulation.

[81] Ho S.Y., Anderson R.H., Sanchez-Quintana D. (2002). Atrial structure and fibres: Morphologic bases of atrial conduction. Cardiovasc. Res..

[82] Zhao J., Butters T.D., Zhang H., Pullan A.J., LeGrice I.J., Sands G.B., Smaill B.H. (2012). An image-based model of atrial muscular architecture: Effects of structural anisotropy on electrical activation. Circ. Arrhythmia Electrophysiol..

[83] Abed H.S., Samuel C.S., Lau D.H., Kelly D.J., Royce S.G., Alasady M., Mahajan R., Kuklik P., Zhang Y., Brooks A.G. (2013). Obesity results in progressive atrial structural and electrical remodeling: Implications for atrial fibrillation. Heart Rhythm.

[84] Azaouagh A., Churzidse S., Konorza T., Erbel R. (2011). Arrhythmogenic right ventricular cardiomyopathy/dysplasia: A review and update. Clin. Res. Cardiol..

[85] Chaumont C., Suffee N., Gandjbakhch E., Balse E., Anselme F., Hatem S.N. (2021). Epicardial origin of cardiac arrhythmias: Clinical evidences and pathophysiology. Cardiovasc. Res..

[86] Ausma J., Wijffels M., Thone F., Wouters L., Allessie M., Borgers M. (1997). Structural changes of atrial myocardium due to sustained atrial fibrillation in the goat. Circulation.

[87] Boyden P.A., Hoffman B.F. (1981). The effects on atrial electrophysiology and structure of surgically induced right atrial enlargement in dogs. Circ. Res..

[88] Neuberger H.R., Schotten U., Verheule S., Eijsbouts S., Blaauw Y., van Hunnik A., Allessie M. (2005). Development of a substrate of atrial fibrillation during chronic atrioventricular block in the goat. Circulation.

[89] Wiegerinck R.F., Verkerk A.O., Belterman C.N., van Veen T.A.B., Baartscheer A., Opthof T., Wilders R., de Bakker J.M.T., Coronel R. (2006). Larger cell size in rabbits with heart failure increases myocardial conduction velocity and QRS duration. Circulation.

[90] Spach M.S., Heidlage J.F., Barr R.C., Dolber P.C. (2004). Cell size and communication: Role in structural and electrical development and remodeling of the heart. Heart Rhythm.

[91] Spach M.S., Heidlage J.F., Dolber P.C., Barr R.C. (2001). Changes in anisotropic conduction caused by remodeling cell size and the cellular distribution of gap junctions and Na(+) channels. J. Electrocardiol..

[92] Dhein S., Hammerath S.B. (2001). Aspects of the intercellular communication in aged hearts: Effects of the gap junction uncoupler palmitoleic acid. Naunyn Schmiedebergs Arch. Pharm..

[93] Dhein S., Seidel T., Salameh A., Jozwiak J., Hagen A., Kostelka M., Hindricks G., Mohr F.W. (2014). Remodeling of cardiac passive electrical properties and susceptibility to ventricular and atrial arrhythmias. Front. Physiol..

[94] De Smet M.A., Lissoni A., Nezlobinsky T., Wang N., Dries E., Perez-Hernandez M., Lin X., Amoni M., Vervliet T., Witschas K. (2021). Cx43 hemichannel microdomain signaling at the intercalated disc enhances cardiac excitability. J. Clin. Investig..

[95] Polontchouk L., Haefliger J.A., Ebelt B., Schaefer T., Stuhlmann D., Mehlhorn U., Kuhn-Regnier F., De Vivie E.R., Dhein S. (2001). Effects of chronic atrial fibrillation on gap junction distribution in human and rat atria. J. Am. Coll. Cardiol..

[96] Verheule S., van Batenburg C.A., Coenjaerts F.E., Kirchhoff S., Willecke K., Jongsma H.J. (1999). Cardiac conduction abnormalities in mice lacking the gap junction protein connexin40. J. Cardiovasc. Electrophysiol..

[97] Kanagaratnam P., Rothery S., Patel P., Severs N.J., Peters N.S. (2002). Relative expression of immunolocalized connexins 40 and 43 correlates with human atrial conduction properties. J. Am. Coll. Cardiol..

[98] Dhillon P.S., Chowdhury R.A., Patel P.M., Jabr R., Momin A.U., Vecht J., Gray R., Shipolini A., Fry C.H., Peters N.S. (2014). Relationship between connexin expression and gap-junction resistivity in human atrial myocardium. Circ. Arrhythmia Electrophysiol..

[99] Rook M.B., van Ginneken A.C., de Jonge B., el Aoumari A., Gros D., Jongsma H.J. (1992). Differences in gap junction channels between cardiac myocytes, fibroblasts, and heterologous pairs. Am. J. Physiol..

[100] Gaudesius G., Miragoli M., Thomas S.P., Rohr S. (2003). Coupling of cardiac electrical activity over extended distances by fibroblasts of cardiac origin. Circ. Res..

[101] Camelliti P., Green C.R., LeGrice I., Kohl P. (2004). Fibroblast network in rabbit sinoatrial node: Structural and functional identification of homogeneous and heterogeneous cell coupling. Circ. Res..

[102] Quinn T.A., Camelliti P., Rog-Zielinska E.A., Siedlecka U., Poggioli T., O’Toole E.T., Knöpfel T., Kohl P. (2016). Electrotonic coupling of excitable and nonexcitable cells in the heart revealed by optogenetics. Proc. Natl. Acad. Sci. USA.

[103] Rubart M., Tao W., Lu X.L., Conway S.J., Reuter S.P., Lin S.F., Soonpaa M.H. (2018). Electrical coupling between ventricular myocytes and myofibroblasts in the infarcted mouse heart. Cardiovasc. Res..

[104] Greisas A., Zlochiver S. (2016). The Multi-Domain Fibroblast/Myocyte Coupling in the Cardiac Tissue: A Theoretical Study. Cardiovasc. Eng. Technol..

[105] Schuessler R.B., Grayson T.M., Bromberg B.I., Cox J.L., Boineau J.P. (1992). Cholinergically mediated tachyarrhythmias induced by a single extrastimulus in the isolated canine right atrium. Circ. Res..

[106] Kneller J., Zou R., Vigmond E.J., Wang Z., Leon L.J., Nattel S. (2002). Cholinergic atrial fibrillation in a computer model of a two-dimensional sheet of canine atrial cells with realistic ionic properties. Circ. Res..

[107] Spach M.S., Dolber P.C., Heidlage J.F. (1989). Interaction of inhomogeneities of repolarization with anisotropic propagation in dog atria. A mechanism for both preventing and initiating reentry. Circ. Res..

[108] Winters J., von Braunmuhl M.E., Zeemering S., Gilbers M., Brink T.T., Scaf B., Guasch E., Mont L., Batlle M., Sinner M. (2020). JavaCyte, a novel open-source tool for automated quantification of key hallmarks of cardiac structural remodeling. Sci. Rep..

[109] Avitall B., Bi J., Mykytsey A., Chicos A. (2008). Atrial and ventricular fibrosis induced by atrial fibrillation: Evidence to support early rhythm control. Heart Rhythm.

[110] Guerra J.M., Everett T.H.t., Lee K.W., Wilson E., Olgin J.E. (2006). Effects of the gap junction modifier rotigaptide (ZP123) on atrial conduction and vulnerability to atrial fibrillation. Circulation.

[111] Tanaka K., Zlochiver S., Vikstrom K.L., Yamazaki M., Moreno J., Klos M., Zaitsev A.V., Vaidyanathan R., Auerbach D.S., Landas S. (2007). Spatial distribution of fibrosis governs fibrillation wave dynamics in the posterior left atrium during heart failure. Circ. Res..

